# No anxiety or pain reduction by Virtual Reality during oocyte retrieval in IVF/ICSI treatment: results of a randomized controlled trial

**DOI:** 10.1093/humrep/deaf193

**Published:** 2025-10-07

**Authors:** A P van Haaps, A M F Schreurs, K Rosielle, V Mijatovic, J W Kallewaard, K Dreyer

**Affiliations:** Department of Reproductive Medicine, Amsterdam University Medical Centers, Location Vrije Universiteit, Amsterdam, The Netherlands; Amsterdam Reproduction and Development, Amsterdam University Medical Centers, Amsterdam, The Netherlands; Department of Reproductive Medicine, Amsterdam University Medical Centers, Location Vrije Universiteit, Amsterdam, The Netherlands; Amsterdam Reproduction and Development, Amsterdam University Medical Centers, Amsterdam, The Netherlands; Department of Reproductive Medicine, Amsterdam University Medical Centers, Location Vrije Universiteit, Amsterdam, The Netherlands; Amsterdam Reproduction and Development, Amsterdam University Medical Centers, Amsterdam, The Netherlands; Department of Reproductive Medicine, Amsterdam University Medical Centers, Location Vrije Universiteit, Amsterdam, The Netherlands; Amsterdam Reproduction and Development, Amsterdam University Medical Centers, Amsterdam, The Netherlands; Department of Anesthesiology, Amsterdam University Medical Centers, Location Academisch Medisch Centrum, Amsterdam, The Netherlands; Department of Anesthesiology, Rijnstate Ziekenhuis, Arnhem, The Netherlands; Department of Reproductive Medicine, Amsterdam University Medical Centers, Location Vrije Universiteit, Amsterdam, The Netherlands; Amsterdam Reproduction and Development, Amsterdam University Medical Centers, Amsterdam, The Netherlands

**Keywords:** Virtual Reality, anxiety relief, pain relief, oocyte retrieval, IVF/ICSI treatment, patient satisfaction

## Abstract

**STUDY QUESTION:**

What is the effect of Virtual Reality (VR) on anxiety and pain during oocyte retrieval in IVF/ICSI treatment?

**SUMMARY ANSWER:**

There is no significant effect of VR on anxiety and pain during oocyte retrieval in IVF/ICSI treatment.

**WHAT IS KNOWN ALREADY:**

Patients undergoing oocyte retrieval in IVF/ICSI treatment often experience anxiety and pain, despite conscious sedation. VR might offer a solution since it has been successful in reducing procedural anxiety and pain during medical procedures, with the potential to replace standard analgesic care.

**STUDY DESIGN, SIZE, DURATION:**

A single-centre, open-label, randomized controlled trial was conducted between February 2023 and August 2024. Due to the nature of the intervention, the study was not blinded.

**PARTICIPANTS/MATERIALS, SETTING, METHODS:**

Patients undergoing oocyte retrieval as part of IVF/ICSI treatment were screened. After providing informed consent, participants were randomized between oocyte retrieval with VR added to conscious sedation and oocyte retrieval with conscious sedation only. When assigned to the intervention group, patients received the VR intervention through a head-mounted device, showing nature films and relaxation exercises. This was added to standard care which includes analgesia and sedatives. Sounds were delivered through the head-mounted device or headphones. The primary outcome was pre- and post-procedural anxiety, measured using the STAI questionnaire. Secondary outcomes included procedural pain (NRS, scale 0–10), satisfaction scores (NRS, scale 0–10), VR preferences, and side effects.

**MAIN RESULTS AND THE ROLE OF CHANCE:**

There were 113 participants included: 57 in the intervention group receiving VR and 56 in the control group not receiving VR. We observed no differences between the intervention and control groups in pre-procedural anxiety (mean difference (MD) 0.14 (95% CI −1.78, 2.05), *P* = 0.885), post-procedural anxiety (MD 0.45 (95% CI −1.21, 2.11), *P* = 0.589), overall pain (MD −0.12 (95% CI −0.97, 0.73), *P* = 0.779), and peak pain (MD 0.59 (−0.51, 1.68), *P* = 0.287).

**LIMITATIONS, REASONS FOR CAUTION:**

VR might only be effective for a certain group of patients undergoing retrieval, or might be more effective in reducing pre-procedural anxiety, which in turn might lead to a reduction in procedural pain. Furthermore, it might reduce pain up to a certain threshold, or be effective when the duration of the procedure is short.

**WIDER IMPLICATIONS OF THE FINDINGS:**

Since VR does not affect anxiety and pain for the general patient population undergoing oocyte retrieval, we do not advise incorporating VR to standard IVF/ICSI anxiety and pain management. For future studies, it is important to investigate which subgroup could benefit from VR and how it could be implemented to study interventions from a non-pharmacological approach. Patient preferences regarding anxiety and pain management during IVF/ICSI treatment should be considered.

**STUDY FUNDING/COMPETING INTEREST(S):**

External funding from ZonMw (Grant number 838002978), the Implementation and Scale-up Coaching, and the Eggcelent Change grant from Theramex have been received for this study to cover the costs of the VR devices. A.P.v.H. and K.R. report to have received a travel grant from Merck to visit ESHRE 2022. A.M.F.S. reports to have been an invited speaker at ESHRE where travel and hotel costs are covered. V.M. reports to have received institutional research grants from Guerbet, Merck, and Ferring. He has received travel and speaker’s fees from Guerbet. J.W.K. reports to be on the Advisory board of Boston Scientific, Saluda, Nevro, Abbott, and Medtronic, and received consulting fees from these organizations. He is a board member of the BNS. KD reports to have received an institutional research grant from Guerbet, a speaker’s fee from Guerbet, and financial support to attend meetings by Merck and Guerbet.

**TRIAL REGISTRATION NUMBER:**

NCT05555498.

**TRIAL REGISTRATION DATE:**

26 September 2022.

**DATE OF FIRST PATIENT’S ENROLMENT:**

7 February 2023.

## Introduction

Infertility is defined by the World Health Organization as the inability to conceive after 1 year of unprotected intercourse and affects one in six couples in the Netherlands (Landelijke netwerkrichtlijn Subfertiliteit, 2010). It can be caused by various factors or remain unexplained. Where female infertility can result from premature ovarian insufficiency, polycystic ovary syndrome, uterine fibroids or endometrial polyps, endometriosis or a tubal factor, male infertility can result from (post-)testicular deficiencies leading to reduced semen quality (Vander Borght and Wyns, 2018; Nik Hazlina *et al.*, 2022). Depending on the cause of infertility, treatment methods like IVF or ICSI can be applied (Allersma *et al.*, 2013; Wang *et al.*, 2019). With IVF/ICSI treatment, multiple mature oocytes (follicles) are induced by performing ovarian stimulation with gonadotropins. The oocytes are collected by aspiration of the follicles through an ultrasound-guided transvaginal puncture, which is known to cause anxiety and pain (Van de Velde, 2005; Allersma *et al.*, 2013; Kwan *et al.*, 2021). Turner *et al.* assessed anxiety levels during all phases of an IVF cycle using the State-Trait Anxiety Inventory (STAI, scale 20–80) and found mean State Anxiety (STAI-S) scores of 41.63 before oocyte retrieval (Spielberger, 1983; Turner *et al.*, 2013). With regards to pain during oocyte retrieval, the recent study by Buisman *et al.* found pain scores between 3.7 and 5.6 (in Numeric Rating Scale (NRS), scale 0–10), depending on the type of pain management (Buisman *et al.*, 2022). Pain is thought to result from needle passage through the vaginal wall, peritoneum, and ovarian capsule, but can be experienced up to several days after oocyte retrieval (Jain *et al.*, 2009; Lier *et al.*, 2015). Both procedural anxiety and pain can negatively affect patient satisfaction which, in turn, is thought to strongly influence therapy compliance, prognosis, utilization of healthcare, and malpractice litigation (Craven *et al.*, 2013; Xesfingi and Vozikis, 2016; Bekelis *et al.*, 2017; Tyser *et al.*, 2018). Moreover, Schaller *et al.* (2016) found that IVF/ICSI treatment caused a significant psychological burden on couples, especially women, leading to premature treatment discontinuation. This underlines the importance of providing adequate psychosocial support throughout treatment and optimizing anxiety and pain management.

There is a large variety of medication protocols that are used for anxiety and pain management in Dutch IVF clinics. The three most frequently used analgesia protocols include intravenously (IV) administered opioids, intramuscularly (IM) administered opioids, and non-sedative oral analgesics. Roest *et al.* found that 61% of IVF clinics have added benzodiazepines to their analgesics to reach a state of conscious sedation where anxiety and pain are sufficiently managed but patients are responsive and able to communicate with the healthcare provider, breathe on their own, and maintain their natural psychological reflexes (Roest *et al.*, 2019). This makes it a relatively effective and safe option. However, anxiety and pain may not be fully alleviated, making an additional non-pharmacological therapeutic options necessary (Vlahos *et al.*, 2009; Frank, 2021; Kwan *et al.*, 2021). Although traditional distraction methods can be helpful, more effective and low-risk alternatives are needed (Hitching *et al.*, 2023). Virtual Reality (VR), initially introduced in 1994 for surgical simulation and medical training, could offer a solution. It distracts the patient by immersing them in a virtual environment resulting in competition between limited shared attentional resources and incoming nociceptive signals. This ultimately leads to reduced (procedural) anxiety and pain, resulting in VR being increasingly used as a successful method to reduce procedural anxiety, and as an adjunct or alternative to pharmacologic analgesia and sedation for a wide range of medical procedures (Chinnock, 1994; Smith *et al.*, 2020; Paro *et al.*, 2022). Studies within the field of gynaecology found it effective in reducing anxiety, pain, or both during hysteroscopy, hysterosalpingography (HSG), episiotomy repair, and intra-uterine device insertion (JahaniShoorab *et al.*, 2015; Chan *et al.*, 2018; Deo *et al.*, 2021; Orhan and Bulez, 2023; Pelazas-Hernandez *et al.*, 2023; Yilmaz Sezer *et al.*, 2023; Oz and Demirci, 2024). Furthermore, Chan *et al.* found that VR significantly reduced anxiety and depressive symptoms, when provided prior to gynaecological procedures (Chan *et al.*, 2018; Zhang *et al.*, 2021).

To our knowledge, the use of VR during oocyte retrieval has not yet been studied (Di Guardo and Palumbo, 2022). Therefore, in this study, we aim to determine the effect of VR on anxiety and pain during oocyte retrieval in IVF/ICSI treatment, thereby enhancing patient satisfaction.

## Materials and methods

### Study design

This randomized controlled trial was performed at the Department of Reproductive Medicine of the Amsterdam UMC location VUmc between February 2023 and August 2024. The study was approved by the Amsterdam UMC institutional review board and prospectively registered at clinicaltrials.gov (reference number NCT05555498).

A sample size was calculated. Our study aimed for an 8-point reduction in anxiety (in STAI, scale 20–80) with an SD of 14, which was based on the study by Turner *et al.* (2013). The desired statistical power was 80% with an alpha of 0.05 and a 10% loss to follow-up rate. This resulted in a calculated sample size of 112 participants. All patients undergoing oocyte retrieval as part of IVF/ICSI treatment were screened for eligibility. We included patients aged between 18 and 43 years old who were undergoing their first, second, or third oocyte retrieval, in the hopes of becoming pregnant. They had to be proficient in English or Dutch language. Patients were excluded if they: used antidepressants, sedatives, or analgesics daily, were undergoing oocyte retrieval because of fertility preservation, had a visual or auditory impairment, suffered from technology-related sensitivity (dizziness, epilepsy, nausea, motion sickness), or were unwilling or unable to provide informed consent. Patients were provided with study information by their treating physician. At least 1 week after, they were provided the information, and patients were contacted by the researcher involved in the study to answer any questions. They were then asked about study participation. Prior to oocyte retrieval, participants were asked to sign their informed consent form, after which they were randomized to the intervention group (oocyte retrieval with conscious sedation and VR) or the control group (oocyte retrieval with conscious sedation but without VR). Randomization was performed by the researcher involved in the study. Participants were told the outcome of randomization prior to their oocyte retrieval. Randomization was performed using Castor (Castor Electronic Data Capture, Ciwit BV, Amsterdam, The Netherlands) in a 1:1 ratio and using variable block sizes of 4–8.

### Study procedures

According to standard local protocol, patients were instructed to arrive at the IVF centre an hour before oocyte retrieval. During this hour, anxiety and pain medication was administered to both the intervention and control group. This consisted of conscious sedation with IM opioids (pethidine or morphine) for pain reduction and oral benzodiazepines (midazolam or diazepam) for anxiety reduction. During the procedure, the participant was placed in a lithotomy position after which the speculum was inserted and the vaginal wall was cleaned. After the speculum was removed, the sterile ultrasound probe with a needle guide was inserted and a hollow needle was inserted into this guide. Under direct ultrasound guidance, the needle punctured the vaginal wall to reach the ovaries. Each follicle was punctured, and the oocyte fluid was aspirated and sent to the laboratory to isolate the oocytes. From start to finish, the procedure usually lasts between 20 and 30 min. Apart from the medical staff, one partner or support person was allowed in the room with the participant.

When participants were assigned to the intervention group, they were given instructions on the VR headset in the hour before retrieval, where they could look through all applications installed on the headset. During the procedure, participants received the eye-covering headset after the vaginal wall was cleaned and speculum removed. Sounds were delivered through the headset itself or headphones connected to the headset depending on personal preference. In case of complaints or adverse events, the headset could be removed. When permitted by the participant, a dedicated researcher was present at all times for technical support with the VR intervention.

### Virtual Reality technology

A CE-certified VR headset was used with software from SyncVR (SyncVR Medical B.V., Utrecht, The Netherlands) installed on the headset. The head-mounted PICO G2 4K device (Pico Interactive Inc., San Francisco, United States of America) included a VR headset and remote control. When participants needed assistance, the researcher was able to take over control of the headset using a handheld device. The SyncVR Relax and Distract software was installed on the VR glasses, which is designed to be used in a medical setting and offers approximately 40 VR interventions. The software includes relaxing nature films and breathing exercises consisting of 3D visualization and accompanying sounds. Participants were able to navigate through the software installed on the VR glasses and choose a nature film or breathing exercise they found most appealing. Although the software is developed for pain management during medical procedures, it was not tailored to oocyte retrieval specifically nor personalized for each individual participant. Interactive games were removed from the VR library to prevent sudden movements during the oocyte retrieval because this was considered undesirable and unsafe for participants. Sounds could be delivered either through headphones connected to the VR glasses or through the VR headset itself. The participants could choose whether they wished to add headphones.

### Outcomes

The primary outcome focused on the difference in anxiety experienced before and immediately after oocyte retrieval (measured using the STAI-S questionnaire). Secondary outcomes included the difference in general anxiety before and 72 h after oocyte retrieval (measured using the STAI-T questionnaire), the difference in procedural pain during oocyte retrieval (in NRS, scale 0–10) between the intervention and control group, which was divided into overall and peak pain scores and pain during the first 72 h after retrieval (in NRS), as well as patient and healthcare provider satisfaction scores (in NRS), experienced side effects, VR expectation, and satisfaction with VR (for the intervention group).

### Data collection

All data were stored using Castor EDC. Baseline characteristics were collected through the electronic patient file where age, duration of infertility, IVF/ICSI cycle number, and vital parameters before and after retrieval were obtained. Healthcare providers were asked to register their procedural satisfaction scores in the patient file, which were also collected. All other data were collected using three sets of questionnaires. Two questionnaires were distributed on paper before and immediately after oocyte retrieval, while the third set was distributed digitally 72 h after the procedure. The first set of questionnaires, distributed before medication administration to minimize confounding bias, included questions about the participants’ VR expectations but also the State Trait Anxiety Inventory-State (STAI-S) and State Trait Anxiety Inventory-Trait (STAI-T) questionnaire. The STAI questionnaires were developed by Spielberger *et al.* and are validated measurement tools to quantify stress (Spielberger, 1983). The STAI-S is used to measure the experienced stress ‘right now’ while the STAI-T questionnaire is used to measure stress ‘in general’. Ultimately, a score between 20 and 80 can be calculated, where a score between 20 and 39 is seen as ‘little perceived stress’, a score between 40 and 59 is ‘average perceived stress’ and a score between 60 and 80 is ‘high perceived stress’. The second set of questionnaires, distributed within 10 min after oocyte retrieval, included the STAI-S to quantify procedural stress. In addition, patients were asked to score their procedural overall pain and peak pain (both in NRS, scale 0–10), their procedural satisfaction (in NRS, scale 0–10), and whether they experienced any side effects. When assigned to the intervention group, participants were also asked about their VR experiences. Finally, the third set of questionnaires was distributed digitally 72 h after oocyte retrieval. It included the STAI-T questionnaire and questions assessing experienced pain and analgesics use in the first 72 h after retrieval.

### Statistical analysis

Data were analysed according to the intention-to-treat principle using IBM SPSS for Windows, version 26.0 (IBM Corp., Armonk, NY, USA). Continuous data were presented with mean and SD for normal distributed data and median with interquartile ranges for non-normal distributed data. Categorical baseline characteristics were presented as absolute numbers and percentages. Differences in anxiety and pain scores between the intervention and control group were studied using the independent samples *t*-test in case of normally distributed data and the Mann–Whitney *U*-test in case of non-normally distributed data. Data distribution was tested by drafting a histogram of the variable in question. Ordinal data were analysed using the Pearson chi-square test. Finally, possible interactions, which were compiled during data analysis, were studied using simple linear regression. These were mostly based on a previous study where we studied the effect of VR on pain during HSG (Rosielle *et al.*, 2024). We studied possible interactions between anxiety, pain and the number of punctured follicles, age, unilateral or bilateral punction, dosage and type of medication, duration of the procedure, IVF/ICSI cycle number, expectations prior to the procedure and VR experiences. Finally, we studied whether there was a possible interaction between the experienced anxiety and procedural pain.

## Results

**Figure 1.**
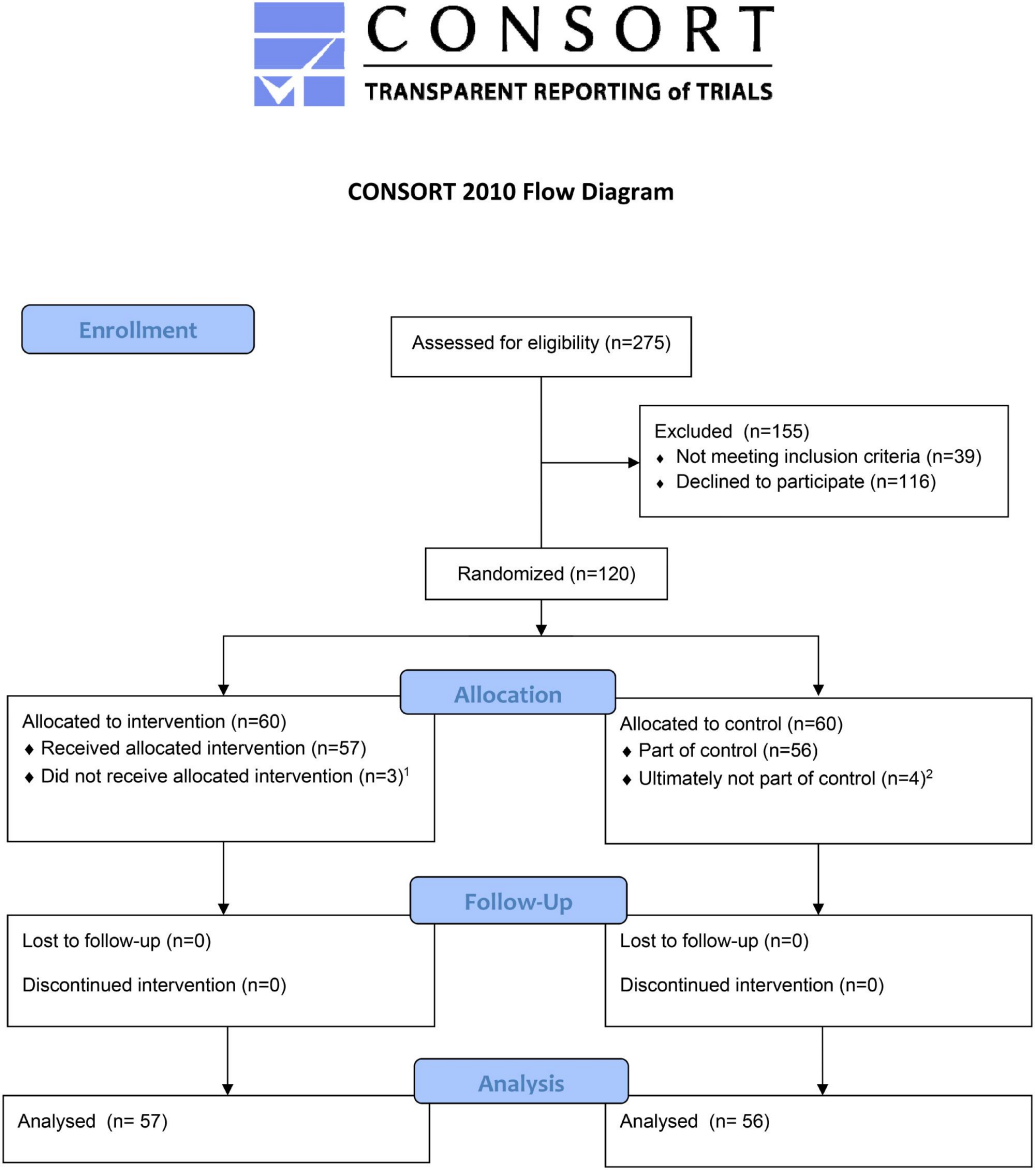
**Flowchart of inclusion**. Patients declined study participation because of previous side-effects with VR interventions (nausea, dizziness, feeling of being seasick), wanting to see the procedure, no additional load by study procedures, or no need of VR intervention. (1) Reasons for not receiving the intervention: withdrew consent after giving randomization results (n=1), thought they also had to sign consent form when they did not want to participate (n=1), went to another hospital (n=1). (2) Reasons for not being part of the control: withdrew consent after giving randomization results (n=2), thought they also had to sign consent form when they did not want to participate (n=1), went to another hospital (n=1).

A total of 275 patients were screened for eligibility, of whom 120 provided informed consent. Ultimately, seven participants did not proceed with oocyte retrieval or withdrew consent after randomization, leading to 57 participants in the intervention group and 56 in the control group being analysed (Fig. 1). Baseline characteristics are presented in Table 1, which showed no significant differences.

**Table 1.** Patient characteristics.

Characteristic	Intervention with VR (n = 57)	Control without VR (n = 56)
Age (year) (mean (SD))	36.18 (3.83)	35.55 (4.05)
BMI (mean (SD))	25.19 (4.37)	23.84 (4.05)
Duration of infertility (years) (mean (SD))	3.70 (2.63)	3.56 (1.56)
Primary infertility (%)	28 (49.1%)	31 (55.4%)
Cause of infertility (%)		
Male infertility	16 (28.1%)	11 (19.6%)
Female infertility	17 (29.8%)	20 (35.7%)
Male and female infertility	5 (8.8%)	5 (8.9%)
Unexplained	19 (33.3%)	20 (35.7%)
Previous fertility treatments^1^		
No previous treatment	13 (22.8%)	14 (25.0%)
Expectant	3 (5.3%)	2 (3.7%)
IUI	31 (54.4%)	37 (66.1%)
Ovulation induction	2 (3.5%)	4 (7.1%)
IVF	11 (19.3%)	7 (12.5%)
ICSI	8 (14.0%)	3 (5.4%)
Type of pain medication used (%)		
Paracetamol	1 (1.8%)	2 (3.6%)
NSAIDs	0 (0.0%)	0 (0.0%)
Opioids (oral)	0 (0.0%)	0 (0.0%)
Anxiolytics		
Midazolam	56 (98.2%)	54 (96.4%)
Diazepam	1 (1.8%)	2 (3.6%)
Opioids (IM)		
Pethidine	56 (98.2%)	54 (96.4%)
Morphine	1 (1.8%)	2 (3.6%)
Dosage of medication used (mean (SD))		
Anxiolytics		
Midazolam (mg)	7.5 (0.00)	7.5 (0.00)
Diazepam (mg)	10.0 (0.00)	10.00 (0.00)
Opioids (IM)		
Pethidine (mg)	137.62 (25.97)	129.17 (22.96)
Morphine (mg)	5.50 (–)	6.75 (0.35)
Paracetamol (mg)	1000 (–)	1000 (0.00)
Number of follicles harvested (mean (SD))^2^	9.05 (6.91)	10.77 (8.37)

1 Some patients had more than one previous treatment method.

2 Range of duration of the procedure was between 3 and 40 min.

VR, Virtual Reality; IM, intramuscular; mg, milligrams.

### Anxiety experienced by patients undergoing oocyte retrieval

Both participants receiving VR and participants not receiving VR reported ‘average perceived stress’ according to the STAI grading system. No significant differences were seen between the intervention and control group in pre- and post-procedural anxiety, measured using the State-Anxiety (STAI-S) (Table 2). Furthermore, no difference was seen in Trait-Anxiety (STAI-T). No significant anxiety reduction in STAI-S or STAI-T, when comparing pre- to post-procedural scores, was seen in either the intervention or control group. The interaction analyses showed no significant influencing factors. No significant correlation was seen between anxiety and overall procedural pain (*R* = 0.010, *P* = 0.305), or between anxiety and worst procedural pain (*R* = 0.03, *P* = 0.08). However, a very weak but statistically significant correlation between post-procedural anxiety and patient satisfaction (*R* = 0.089, *P* = 0.002) was seen (Supplementary Figs S1, S2 and S3).

**Table 2.** Difference in anxiety, measured using the State-Anxiety and Trait-Anxiety, calculated between women receiving and not receiving VR during oocyte retrieval.

Outcome	Intervention with VR (n = 57)	Control without VR (n = 56)	Mean difference (95% CI)	***P*-value** ^1^
Pre-procedural anxiety (STAI-S) ^2^ (mean (SD))	43.61 (4.15)	43.75 (5.82)	0.14 (−1.78, 2.05)	0.885
Post-procedural anxiety (STAI-S) ^2^ (mean (SD))	45.43 (3.89)	45.88 (4.64)	0.45 (−1.21, 2.11)	0.589
Anxiety reduction STAI-S (mean (SD))^3^	−1.75 (5.37)	−1.78 (5.71)	−0.03 (−2.22, 2.15)	0.975
General pre-procedural anxiety (STAI-T)^2^ (mean (SD))	45.68 (4.65)	44.92 (3.45)	−0.75 (−2.31, 0.81)	0.340
General post-procedural anxiety (STAI-T)^2^ (mean (SD))	45.16 (3.33)	44.78 (3.51)	−0.39 (−1.89, 1.12)	0.611
Anxiety reduction STAI-T (mean (SD))^4^	0.42 (3.57)	0.51 (2.74)	0.09 (−1.34, 1.53)	0.893

State-Anxiety: STAI-S; ‘how are you feeling right now’.

Trait-Anxiety: STAI-T; ‘how are you feeling in general’.

Anxiety was measured both before and after the procedure.

1 Calculated using the independent samples *t*-test.

2 Score between 20 and 39 is considered ‘little perceived stress’, a score between 40 and 59 as ‘an average amount of stress’ and a score between 60 and 80 as ‘high perceived stress’.

3 Calculated by deducting the post-procedural STAI-S score from the pre-procedural STAI-S score.

4 Calculated by deducting the post-procedural STAI-T score from the pre-procedural STAI-T score.

VR, Virtual Reality; STAI, State-Trait Anxiety Inventory.

### Procedural pain during oocyte retrieval

**Figure 2.**
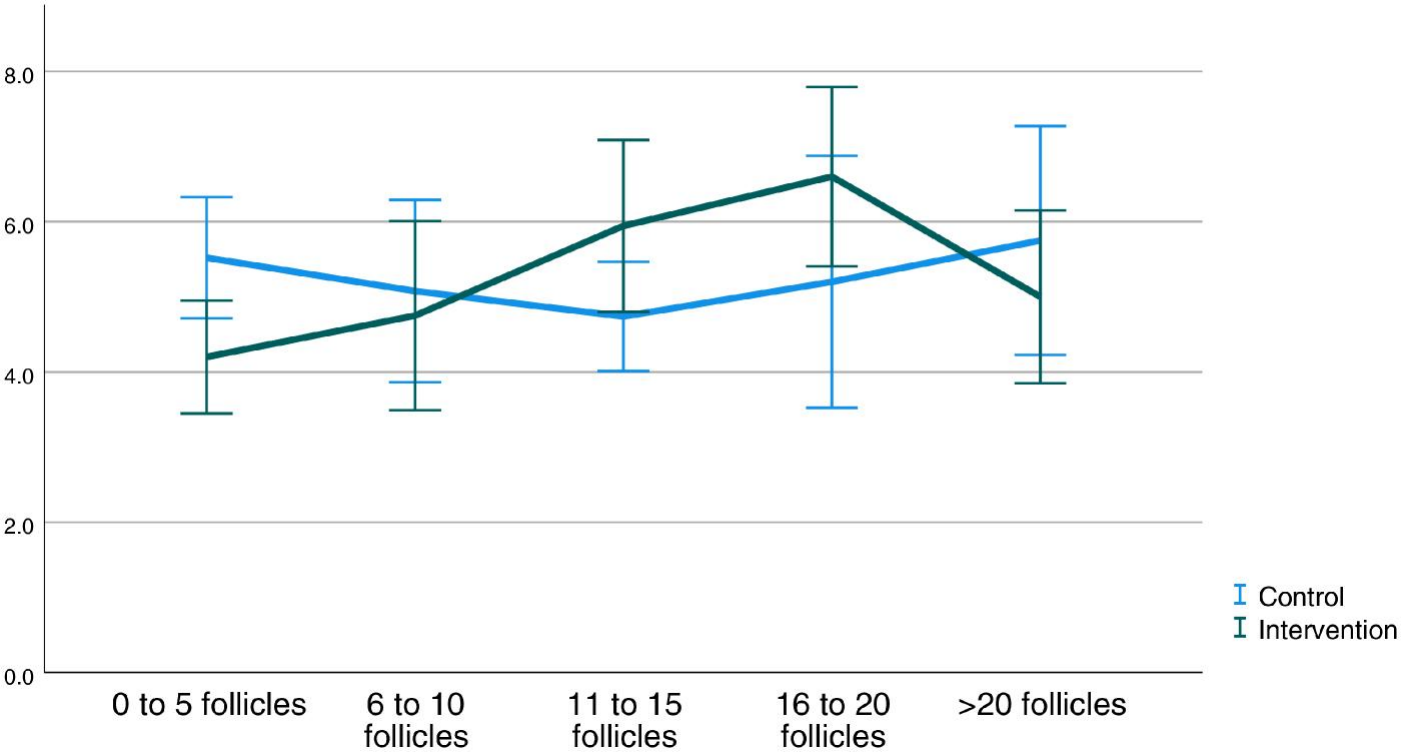
Association between number of follicles harvested during oocyte retrieval and average reported pain scores.

No significant differences in overall procedural pain, peak pain, and time spent thinking about pain were seen between participants receiving VR and participants not receiving VR (Table 3). When performing an interaction analysis, a near-significant interaction was found between overall pain and the number of follicles punctured (*P* = 0.055). Figure 2 showed that this could be attributed to the difference seen when 10 follicles or fewer were punctured. When correcting for the number of follicles (i.e. 10 follicles or less punctured), we saw a significant reduction in overall pain when comparing participants receiving VR to participants not receiving VR (mean difference (MD) 0.93 (95% CI 0.04, 1.83), *P* = 0.041; Table 3). Age distribution was not different between the two groups (MD 1.02 (95% CI −0.48, 2.52), *P* = 0.181). All other potentially influencing factors (dosage of opioids, age, anxiety scores, VR expectations, duration of the procedure, unilateral or bilateral punction) showed no significant differences.

**Table 3.** Difference in time spent thinking about pain, in overall and worst pain scores during oocyte retrieval.

Outcome	Intervention with VR (n = 57)	Control without VR (n = 56)	Mean difference (95% CI)	***P*-value** ^1^
Overall procedural pain (VAS) (mean (SD))	4.00 (1.81)	3.88 (1.39)	−0.12 (−0.97, 0.73)	0.779
Peak procedural pain (VAS) (mean (SD))	5.05 (2.16)	5.64 (2.10)	0.59 (−0.51, 1.68)	0.287
Time spent thinking about pain (mean (SD))^2^	3.21 (1.08)	3.13 (1.08)	−0.08 (−0.49, 0.33)	0.695

Outcome	10 follicles or less (n = 64)	>10 follicles (n = 48)	Mean difference (95% CI)	*P*-value^1^
Duration of the procedure (min) (mean (SD))	12.72 (7.60)	20.83 (7.79)	−8.11 (−11.02, −5.21)	<0.001

Interactions^3^	Unstandardized coefficients (B) with 95% CI	*P*-value^3^
Overall procedural pain scores and number of harvested follicles^4^	−0.08 with 95% CI (−0.17, 0.002)	0.055
Peak procedural pain scores and number of harvested follicles	−0.04 with 95% CI (−0.14, 0.05)	0.378

Outcome^5^	Intervention with VR (n = 37)	Control without VR (n = 32)	Mean difference (95% CI)	*P*-value^1^
Overall procedural pain (VAS) (mean (SD))	4.41 (1.91)	5.34 (1.80)	0.93 (0.04, 1.83)	0.041

This was calculated between women receiving VR during oocyte retrieval, and women not receiving VR during oocyte retrieval.

1 Calculated using the independent samples *t*-test.

2 Asked on a 5-point Likert Scale (range 1–5, from ‘not at all’ to ‘completely/all the time’).

3 Subgroup analyses with interaction between procedural pain scores and number of harvested follicles were studied, calculated using simple linear regression.

4 Age distribution did not differ between the two groups (mean difference of 1.02 (95% CI −0.48, 2.52), *P* = 0.181).

5 Difference in overall procedural pain in retrievals where 10 follicles or less were punctured.

VR, Virtual Reality; VAS, Visual Analogue Score.

### Procedural patient satisfaction and VR experiences

Procedural satisfaction scores reported by healthcare providers showed no significant differences. Although patient satisfaction scores were high both for participants receiving VR (8.99 (1.04)) and participants not receiving VR (9.34 (0.80)), we did see that participants receiving VR reported significantly lower satisfaction scores (MD 0.35 (95% CI 0.001, 0.702), *P* = 0.050; Table 4). In contrast, participants who underwent oocyte retrieval with VR were significantly less willing to undergo another retrieval without VR (MD 0.86 (95% CI 0.38–1.33), *P* < 0.001), more willing to undergo another retrieval with VR (MD −1.23 (95% CI −1.61 to −0.85), *P* = 0.031) and more likely to recommend VR to others (MD −0.55 (−1.05 to −0.06), *P* < 0.001). When performing interaction analyses, we saw that the IVF/ICSI cycle number and VR expectations prior to the procedure did not influence satisfaction scores or the willingness to undergo another retrieval with or without VR. Procedural side effects did not differ significantly among the two groups (Table 4). The VR experience provided moderate to high distraction (mean 3.55 (SD 0.96) on 5-point Likert scale), and participants experienced moderate immersion in the VR environment (mean 3.11 (SD 1.10) on 5-point Likert scale). A total of 16 participants reported that their expectations were only partially met, with their reasons described in Table 5. Furthermore, reasons for VR removal are described in Table 5.

**Table 4.** Procedure satisfaction reported by the patient and healthcare provider, and experienced side effects.

Procedural satisfaction immediately after oocyte retrieval	Intervention with VR (n = 57)	Control without VR (n = 56)	Mean difference (95% CI)	***P*-value** ^1^
Procedural satisfaction score patient^3^ (mean (SD))^2^	8.99 (1.04)	9.34 (0.80)	0.35 (0.001, 0.702)	0.050
Procedural satisfaction score healthcare provider^3^ (mean (SD))^2^	8.46 (1.74)	8.14 (1.44)	−0.32 (−1.03, 0.40)	0.378
Another oocyte retrieval with VR^4^ (mean (SD))^1^	4.29 (1.26)	3.74 (1.35)	−1.23 (−1.61, −0.85)	0.031
Another oocyte retrieval without VR^4^ (mean (SD))^1^	3.35 (1.42)	4.21 (1.08)	0.86 (0.38, 1.33)	<0.001
Recommend VR during oocyte retrieval to others^4^ (mean (SD))^1^	4.39 (0.82)	3.16 (1.10)	−0.55 (−1.05, −0.06)	<0.001
Side effects^4^ (mean (SD))				
Nausea	1.33 (0.81)	1.24 (0.73)		0.525
Dizziness	1.81 (0.97)	2.10 (1.07)		0.142
Headache	1.11 (0.56)	1.04 (0.19)		0.393
Blurred vision	1.88 (1.15)	2.10 (1.27)		0.345

Differences were calculated between women receiving VR during oocyte retrieval, and women not receiving VR during oocyte retrieval.

1 Calculated using the Pearson chi-square test.

2 Calculated using the independent samples *t*-test.

3 Asked to score their satisfaction on a scale from 1 to 10.

4 Asked on a 5-point Likert Scale (range 1–5, from ‘not at all’ to ‘completely/all the time’).

VR, Virtual Reality.

**Table 5.** Patient experiences with the VR intervention.

VR experiences	Intervention with VR (n = 57)
Headphones worn during the procedure (%)	9 (15.8%)
Pain reduction by VR^3^ (mean (SD))	3.30 (1.14)
Distracted by VR^3^ (mean (SD))	3.55 (0.96)
Able to concentrate on VR^3^ (mean (SD))	3.18 (1.04)
Ability to immerse in VR environment^3^ (mean (SD))^1^	3.11 (1.10)
Satisfaction with VR applications^3^ (mean (SD))	3.63 (1.00)
VR expectations were met (%)	
Yes	40 (70.2%)
Partially	16 (28.0%)
No	0 (0.0%)
Unknown	1 (1.8%)
Reasons expectations were partially met (%)^2^	
Insufficient distraction	9 (15.8%)
Insufficient pain reduction	1 (1.8%)
Insufficient stress reduction	2 (3.5%)
Confusing to focus on healthcare provider and VR	3 (5.3%)
Blurry images	3 (5.3%)
Technical	2 (3.5%)
Unknown	1 (1.8%)
VR headset removed during the procedure (%)	
Wanting to see what happens	2 (3.5%)
Too much procedural pain	2 (3.5%)
VR headset malfunction	2 (3.5%)
Too sleepy from medication	1 (1.8%)

1 When performing a confounding analysis, no influence of VR immersiveness was seen by whether the patient wore headphones during the procedure or not.

2 Some women gave more than one reason why VR expectations were only partially met.

3 Asked on a 5-point Likert Scale (range 1–5, from ‘not at all’ to ‘completely/all the time’).

VR, Virtual Reality.

### Pain and patient satisfaction 72 h after oocyte retrieval

In the first 72 h after oocyte retrieval, overall and peak pain scores did not show a significant difference between the intervention and control group. Patient experience of oocyte retrieval was equal in the intervention and control group. With regards to undergoing another oocyte retrieval with and without the VR intervention, as well as recommending them to others, we observed the same results as we did immediately following the procedure. Participants not receiving VR reported significantly more dizziness, headache, and blurred vision than participants receiving VR in the first 72 h after retrieval (Table 6).

**Table 6.** Pain scores, procedural satisfaction and experienced side effects 72 h after oocyte retrieval in IVF/ICSI treatment.

72 h after oocyte retrieval (mean (SD))	Intervention with VR (n = 57)	Control without VR (n = 56)	Mean difference (95% CI)	***P*-value** ^1^
Overall pain (VAS) (mean (SD))	4.05 (1.83)	4.16 (1.39)	0.11 (−0.67, 0.90)	0.776
Peak pain (VAS) (mean (SD))	5.12 (2.20)	5.77 (2.01)	0.65 (−0.36, 1.66)	0.201

**Procedural satisfaction** ^2^	**Intervention with VR (n = 57)**	**Control without VR (n = 56)**	**Mean difference** **(95% CI)**	***P*-value** ^1^

Experience of oocyte retrieval (mean (SD))	3.50 (0.90)	3.42 (0.95)	−0.08 (−0.48, 0.32)	0.694
Another oocyte retrieval with VR (mean (SD))	4.35 (0.96)	3.42 (1.24)	0.97 (0.53, 1.40)	<0.001
Another oocyte retrieval without VR (mean (SD))	3.38 (1.08)	4.34 (0.91)	−0.93 (−1.42, −0.45)	<0.001
Recommend VR during oocyte retrieval to others (mean (SD))	4.35 (0.76)	3.08 (0.88)	−1.28 (−1.63, −0.92)	<0.001
Side effects (mean (SD))				
Nausea	1.54 (0.97)	1.76 (1.13)		0.065
Dizziness	1.60 (0.87)	2.05 (0.93)		0.044
Headache	1.42 (0.68)	1.61 (1.03)		0.047
Blurred vision	1.33 (0.60)	1.87 (1.04)		0.033
Tiredness	3.08 (1.05)	3.53 (1.06)		0.303
Concentration problems	2.04 (0.97)	2.61 (1.18)		0.112

Differences were calculated between women receiving VR during oocyte retrieval, and women not receiving VR during oocyte retrieval.

1 Calculated using the Pearson chi-square test.

2 Questions were asked on a 5-point Likert Scale (range 1–5, from ‘not at all’ to ‘completely/all the time’).

VR, Virtual Reality; VAS, Visual Analogue Score.

## Discussion

This is the first study investigating the effect of VR on anxiety and pain during oocyte retrieval. We found no significant differences in anxiety, overall, and peak pain between participants receiving VR and participants not receiving VR. We found no significant anxiety reduction when comparing pre- to post-procedural scores. Participants receiving VR reported significantly lower procedure satisfaction scores immediately after oocyte retrieval but were significantly more inclined to undergo another retrieval with VR and more likely to recommend VR during retrieval to others.

Previous studies on the use of VR during gynaecological procedures are contradictory. Where some found no effect of VR on anxiety, pain, and patient satisfaction during HSG and colposcopy, others did find it effective in similar gynaecological procedures like hysteroscopy, HSG, amniocentesis, and episiotomy repair (JahaniShoorab *et al.*, 2015; Deo *et al.*, 2021; Melcer *et al.*, 2021; Hecken *et al.*, 2023; Orhan and Bulez, 2023; Pelazas-Hernandez *et al.*, 2023; Yilmaz Sezer *et al.*, 2023; Rosielle *et al.*, 2024). Additionally, strong correlations between anxiety and pain have been suggested. Severe anxiety was associated with an increased demand for pain medication and pre-operative anxiety has been found a predictor of postoperative pain in hysterectomy patients (Craven *et al.*, 2013; Zhang *et al.*, 2021). In our study, we primarily provided VR during the procedure, which could explain why we found no effect on anxiety and pain. Since VR was only shortly provided during the waiting time, with the aim of trying out the intervention rather than reducing pre-procedural anxiety, it might have been suboptimal in reducing pre-procedural anxiety and thereby procedural pain. For future studies, the effect of VR on pre-procedural anxiety and consequently on post-procedural pain should be further investigated.

In addition, VR might only be effective up to a certain pain threshold but when pain exceeds this threshold or the procedure takes longer, pain management might become insufficient. van Barneveld *et al.* (2023) found that the reporting of overall pain scores was influenced by recall bias when patients were asked to retrospectively score their pain. It resulted in them overestimating pain intensity. In our study, it might be that the oocyte retrieval was manageable in terms of pain at first, but intensified after a certain time, making distraction of the VR intervention and its effect on pain no longer sufficient. Therefore, participants might have scored based on this more intense pain, already forgetting the manageable pain at the start of the procedure, leading to a possible overestimation. In our interaction analysis, we found significantly lower overall pain scores in the intervention group when 10 follicles or less were punctured. These retrievals were found significantly less time-consuming and less aspiration attempts were often needed to collect all follicles. This could suggest that small retrievals are associated with less pain, but it might also be that overall pain scores were less affected by recall bias as the duration of the procedure was significantly shorter. Nevertheless, since we did not base our sample size on this finding, we cannot attribute this effect on pain scores to VR with full certainty.

VR has been proven to be a safe treatment method with minor and short-term side effects. In our study, we found that participants in the intervention group experienced less side effects such as dizziness, headache, and blurred vision in the first 72 h after retrieval, compared to participants in the control group. This might be due to the fact that VR has been proven successful as vestibular rehabilitation (a therapy to manage dizziness, headaches, and balance issues) in case of vertigo (Xie *et al.*, 2021). Nonetheless, despite generally high patient satisfaction, participants in the intervention group reported significantly lower satisfaction scores. This could be partly explained by technical difficulties with the VR intervention at times, but it is also possible that it resulted from unmet expectations of VR. Participants whose expectations were only partially met, attributed this to the fact that VR provided them insufficient distraction, or pain or anxiety reduction. Interestingly, however, participants receiving VR were significant more willing to undergo another retrieval with VR, and less willing to undergo another retrieval without VR. It is possible that, although the VR intervention has not been proven optimally effective, participants still experienced a sense of control regarding their anxiety and pain management. Previous studies found that improved self-management skills had a positive effect on quality of life and patient satisfaction, in patients diagnosed with chronic disorders (Musekamp *et al.*, 2016; Li and Hapidou, 2021; van Haaps *et al.*, 2023). This is similar to findings in our previous study, where we applied VR during HSG. We found no effect on overall and peak pain scores, but did find that patients receiving VR during HSG were significant more willing to undergo another HSG with VR, and less willing to undergo another HSG without it (Rosielle *et al.*, 2024). It might therefore be that although VR objectively did not provide anxiety and pain relief and the participants’ expectations were not always fully met, they might still have perceived VR as a valuable tool to offer some level of distraction or comfort, making them prefer it in future procedures. Furthermore, it might have offered participants a sense of control or provided an additional coping mechanism. This might have made them feel more empowered or better equipped to handle the procedure, leading to more willingness to undergo the procedure again with VR despite lower satisfaction. Patient satisfaction is considered important, as studies linked it to better medication adherence, better treatment outcomes, lower healthcare costs, and improved health-related quality of life (Renzi *et al.*, 2005; Dubina *et al.*, 2009; Durmus and Akbolat, 2020). There is a growing recognition that innovations in treatment should focus on more than just pain relief, and healthcare providers emphasize the importance of adopting a holistic approach. This approach addresses a spectrum of health domains and assesses patients’ unmet needs at baseline while tracking treatment-induced improvements across these domains (Levy *et al.*, 2023). As a result, patient satisfaction could be considered an important outcome measure, potentially outweighing pain and/or anxiety reduction.

In future studies, it might be interesting to investigate whether VR could be further tailored as generic VR interventions might not achieve the optimal desired effect. Not all patients might react similarly, and might want to gain different things from the VR intervention or might need different stimuli in order to achieve optimal anxiety and pain management, making it interesting to add patient preferences when developing new VR software. Pardini *et al.* performed a pilot study and found that a personalized VR intervention was more effective in reducing patient-reported anxiety and tension, compared to a standard VR intervention. They stated that further research was needed (Pardini *et al.*, 2024). In addition, it could be interesting to include which subgroup of patients will benefit from a VR intervention and when they will benefit most. It is possible that patients of older age might respond differently to the VR intervention than those of younger age, where the VR devices could be more effective in optimizing positive emotions in younger patients (Pavic *et al.*, 2024). Nevertheless, in our study, we saw no significant differences in age distribution between our groups and age itself was no influencing factor. However, the interaction analysis did suggest that participants undergoing smaller and quicker retrievals benefitted more from the VR intervention, which might suggest that VR is most effective when applied during shorter gynaecological procedures with lower baseline pain scores. Furthermore, it is also possible that VR is more effective in reducing pre-procedural anxiety and thereby influence procedural pain. Finally, it can be questioned whether the infertile patient is the right patient to provide VR to. They have already had to change their expectations and have experienced a loss of control over when and how they become pregnant, but do have control over how they undergo oocyte retrieval. The fact that about 50% of eligible patients declined study participation, with the most frequent reason being that they wanted to see what happened, keep control and/or wanted to keep in contact with the healthcare provider, could also be an indication of this (Supplementary Table S1). Some patients who declined participation mentioned that they felt the retrieval was the closest they came to conceiving a child together, making that they wanted to be present during the procedure. Therefore, it is important to further explore patient preferences by in-depth interviews or analysing qualitative feedback, to understand their views on anxiety and pain management during IVF/ICSI treatment.

### Strengths and limitations

Our study has several strengths. First, we added sound to the VR environment to optimize VR immersiveness, an important factor in reducing pain and/or anxiety (Matamala-Gomez *et al.*, 2023). Also, because in our previous study where VR was provided during HSG, we saw that adding sound might improve the efficacy of VR and participants reported missing sound from the VR intervention with a mean score of 2.5 (SD 1.30), on a 5-point Likert scale (Rosielle *et al.*, 2024). Furthermore, all eligible patients were counselled by the same researcher to ensure consistency of information. Finally, we performed interaction analyses to explore whether VR was more effective for certain patient groups. Our study also has several limitations. First, a significant number of patients declined to participate in the study (112/232), with frequently mentioned reasons that participants wanted to see and hear what was happening. This could have introduced selection bias. In addition, healthcare providers had to communicate with the patient at times, potentially leading to reduced VR immersiveness. Furthermore, all participants received conscious sedation, which might have contributed to suboptimal VR immersiveness. Since they were somewhat dazed from medication, they might have been unable to focus on the VR intervention optimally. However, no studies support this statement, and with the exception of one participant, we often saw that patients were very alert. In addition, the VR intervention was not tailored to the procedure or the specific patient preferences, although patients were able to choose which nature film or relaxation exercise was most appealing to them. Therefore, the generic VR intervention might not have achieved optimal pain and anxiety management. However, at the time of initiation of our study, personalization of the VR intervention was not available. Additionally, since both the participant and researcher dedicated to the trial were not blinded, this might have contributed to performance bias. Furthermore, a total of seven participants removed the VR headset, with reasons described in Table 5. Finally, we did not objectify anxiety after the VR intervention was provided in the waiting time prior to the procedure. It might be that VR is more effective in reducing pre-procedural anxiety and thereby procedural pain. This should be further assessed in future studies.

## Conclusion

We did not find an effect of VR on anxiety and procedural pain. However, the interaction analysis suggested VR might be effective in reducing overall pain for participants undergoing a retrieval where 10 or fewer follicles were punctured. However, since we did not base our sample size on this finding, we cannot attribute this to VR with full certainty. Nonetheless, it might suggest that VR only reduces pain up to a certain threshold and for a certain period of time, but is no longer effective when it exceeds this. Furthermore, it is important to investigate which subgroup could benefit from VR and would want to implement this in their anxiety and pain management. Therefore, patient preferences are an important outcome measure and patient views on anxiety and pain management during IVF/ICSI treatment should be explored. The infertile patient might not be the right patient to provide VR to. We saw that there was a great need for control during the puncture among patients. In conclusion, based on the results of this study, we do not advise incorporating VR during oocyte retrieval in standard IVF/ICSI anxiety and pain management.

## Supplementary Material

deaf193_Supplementary_Figure_S1

deaf193_Supplementary_Figure_S2

deaf193_Supplementary_Figure_S3

deaf193_Supplementary_Table_S1

## Data Availability

The data underlying this article will be shared on reasonable request to the corresponding author.
