# Supplementary material for: No anxiety or pain reduction by Virtual Reality during oocyte retrieval in IVF/ICSI treatment: results of a randomized controlled trial
Source: Hum Reprod. 2025 Oct 7;41(1):39–49. doi: 10.1093/humrep/deaf193 (PMC12769444; doi:10.1093/humrep/deaf193)
Supplement: deaf193_Supplementary_Figure_S3 [file deaf193_supplementary_figure_s3.pdf]

Human Reproduction, 2025, 1–1
https://doi.org/10.1093/humrep/deaf193
Supplementary Data

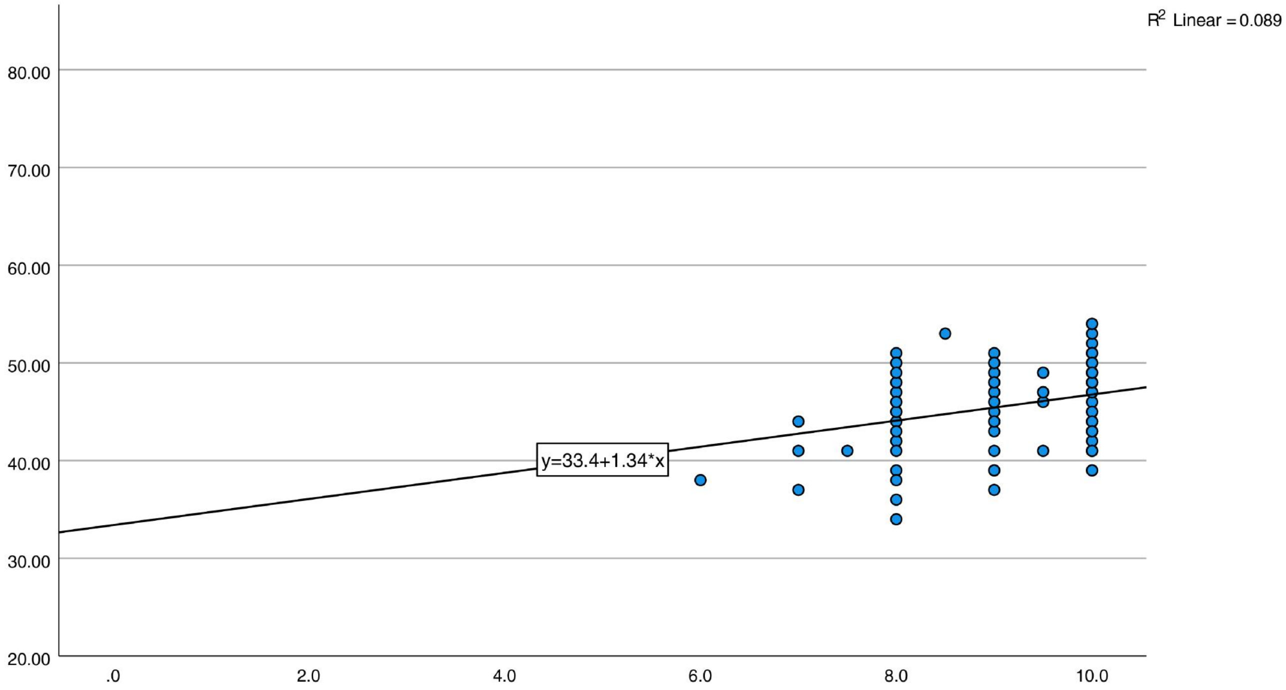

Supplementary Figure S3. Correlation between patient satisfaction scores (in NRS, scale 0–10) and post-procedural anxiety (STAI-S, scale 20–80).
