# Supplementary material for: No anxiety or pain reduction by Virtual Reality during oocyte retrieval in IVF/ICSI treatment: results of a randomized controlled trial
Source: Hum Reprod. 2025 Oct 7;41(1):39–49. doi: 10.1093/humrep/deaf193 (PMC12769444; doi:10.1093/humrep/deaf193)
Supplement: deaf193_Supplementary_Table_S1 [file deaf193_supplementary_table_s1.pdf]

OXFORD
human reproduction
Human Reproduction, 2025, 1–1
https://doi.org/10.1093/humrep/deaf193
Supplementary Data

Supplementary Table S1. Reasons for study decline.

Reason for declining study participation	Number of declined participants (n = 116)
General refusal	69 (59.4%)
Wants to see what is happening, keep contact with the healthcare provider and/or keep control	16 (13.8%)
Does not need additional anxiety and/or pain management	12 (10.3%)
Does not want anything extra since the procedure was already stressful	10 (8.6%)
Previous negative experience with VR, does not want to trigger their cluster headache	6 (5.2%)
Wants to try other relaxation and pain managing options (e.g. pelvic floor relaxation)	1 (0.9%)
Too time consuming	1 (0.9%)
Not mentioned	1 (0.9%)

© The Author(s) 2025. Published by Oxford University Press on behalf of European Society of Human Reproduction and Embryology.
This is an Open Access article distributed under the terms of the Creative Commons Attribution License (https://creativecommons.org/licenses/by/4.0/), which permits unrestricted reuse, distribution, and reproduction in any medium, provided the original work is properly cited.
Human Reproduction, 2025, 00, 1–15
https://doi.org/10.1093/humrep/deaf193
Original Article
